# Detrimental effects of scene manipulations on temperature-based time since death estimation

**DOI:** 10.1007/s00414-024-03252-w

**Published:** 2024-05-21

**Authors:** Patrick Sauer, Constantin Lux, Hannes Gruber, Marcel A. Verhoff, Frank Ramsthaler, Natascha Kern, Mattias Kettner

**Affiliations:** 1 Institute of Legal Medicine, University Hospital of Frankfurt, Goethe University, Kennedyallee 104, 60596 Frankfurt/Main, Germany; 2 Institute of Legal Medicine, University Hospital of Homburg/Saar, University of Saarland, Geb. 49.1, Kirrberger Straße, 66421 Homburg/Saar, Germany

**Keywords:** Post mortem interval, Nomogram method, Crime scene, Ambient temperature change, Ventilation heat loss, Window opening

## Abstract

In forensic casework, time since death (TSD) estimations may play a crucial role to establish chains of events as well as for alibi assessment in homicide cases. Classical TSD estimation relies on reasonably stable ambient temperatures and a correct documentation of ambient and rectal temperatures. This constancy is in some cases disturbed by post-discovery alterations of the crime scene, e.g. opening a window. In order to develop a better understanding of this alteration-based detrimental impact on TSD estimation as well as to identify feasible recommendations for casework, the present pilot study examined ambient temperature effects of different window opening scenarios regarding various time intervals (5 to 360 min) in a furnished 10 m^2^ apartment during winter. In this context, in addition to the ambient temperature and thus the cooling rate of the room, re-approximation to initial room temperature, potential influences on a nomogram-based time since death estimation using a fictitious case, and the impact of the measurement height above the ground were investigated. Our data indicate a significant reduction of the mean temperature decrease rate after 15 min regardless of the remaining opening time and a correlation with the size of the respective opening surfaces. Re-approximation to initial room temperatures was observed with up to three times longer than the initial opening time. There was no evidence of a substantial advantage of temperature measurements above the level of the corpse (> 0.1 m). The limitations of the study and its applicability for forensic casework are critically reviewed.

## Introduction

The estimation of the time since death (TSD) plays a crucial role in medicolegal casework especially concerning unobserved homicides, cases in which witness accounts are inconsistent or not deemed trustworthy, cases of failures to render first aid, or cases in which the time of death is decisive for civil claims (e.g. determination of succession). TSD estimations are performed to narrow down the time interval in which death occurred, e.g., in order to reduce potential suspect numbers, focus investigation efforts, and assess alibis. At the onset of every criminal investigation of a corpse discovery site the development of a general hypothesis, concerning the rough time frame of death is a useful instrument to focus the investigative work (e.g., interrogation of potential witnesses regarding time sequences and relations). TSD estimations employ a wide spectrum of information derived from the examination of the corpse and its surroundings as well as the analysis of samples (e.g. maggots for entomological examinations) acquired during inspection or autopsy. The method(s) of choice for early or later stages of TSD may include assessment of rigor and livor mortis, the alignment of the body temperature with the ambient temperature (in most instances body cooling due to lower ambient temperature), supravital reactions like the mechanical and electrical excitability of muscle or chemical methods (“so called” Compound method), assessment of the putrefaction stage as well as entomological methods [1–3]. Albeit that at least some scientific attention has been drawn to all of these criteria [4–9] the main focus of more recent research efforts has been on temperature-based methods of TSD estimation or derivatives thereof.

The method of Henssge is the current gold standard of temperature based TSD estimation [1, 10] in the early postmortem period. It is in itself based on the mathematical description of body cooling by Marshall and Hoare [11–13]. As a practical solution for crime scene use, nomograms have been provided by Henssge, which led to the method to be commonly known as the Nomogram method [14–17]. The Nomogram method utilizes ambient temperature, rectal temperature, and body weight as well as corrective factors (or correction factors) to be executed on body weight before calculation to establish an estimate of the TSD in the form of a 2 standard deviations death time interval. The left side of the underlying mathematical equation displays the so-called Q-value, which results from the difference between the measured rectal temperature at the time of investigation and the ambient temperature divided by the difference between the assumed body core temperature at the beginning of body cooling (37.2 °C) and the measured ambient temperature. In simplified terms, the Q-value can be considered as a mathematical expression of the “cooling capacity” of the corpse starting at 1.0 (or 100%) after death decreasing up to the point of complete temperature equalization with the environment. The calculation of the TSD is only feasible until the so-called critical Q-value is reached (0.2 for ambient temperatures < 23.3 °C or 0.5 for ambient temperatures of ≥ 23.3 °C), as from that point on even small variations in the measured temperature values (e.g. measurement inaccuracy of the utilized thermometer [18]) can result in large and therefore erroneous death time intervals.

In recent years the classical Nomogram method has been subjected to numerous alteration attempts and additional features to refine the results of the method have been developed, e.g., a nomogram integrating correction factors for bodyweight [19]. While the results obtained by the Nomogram method are rather robust, the resulting death time intervals have been criticized for being too wide. This problem was addressed using the “so called” Compound method [20–22].

Additional methodological approaches, e.g., numerical simulations of the underlying physics [23–25] and brute force calculation of parameters [26, 27] have been suggested. Furthermore, variations in standard baseline parameters (such as a standardized body core temperature at the time of death or a given ambient temperature) were examined, e.g., for hyper- and hypothermia case constellations [28–30] and for case constellations, in which the ambient temperature is unknown [31]. In addition, mathematical approaches to describe changes of ambient temperature were published [32–34]. Numerous scientific studies have revealed, that the ambient temperature (and the actual initial body temperature at the time of death) are suspected to be the most significant contributors to errors in temperature-based TSD estimations [35–37]. For instance Weiser et al. pointed out, that an inaccurate characterization of the environmental temperature rate (environmental temperature change since the time of death) could possibly lead to 10–20% estimation error and more [36]. Due to the significance as a source of error, some studies have examined modulation attempts without including the ambient temperature [31, 38].

Most TSD estimation methods, however, assume that parameters remain relatively permanent. While this permanence is partially self-evident, e.g., as pertains to the body weight, other parameters may show considerable variations either in the pre-detection phase or after discovery of the body (such as air flow [39] and ambient temperature). Crime scenes inside of buildings, which are constructed as a form of shelter, compared to outdoor crime scenes, can generally be assumed as more stable with regards to temperature, air flow and humidity. In a typical given case scenario, the ambient temperature is usually considered to be relatively stable during the post-detection phase until temperature measurements are performed. This usually holds true for the scene circumstances onward from the moment measurements are taken, since at this point in the investigation process, specialists of the criminal police have taken over, which should be aware of the necessity for stable temperature conditions. This permanence is then upheld by minimizing alterations of the surroundings of the scene. In some cases, however, surrounding conditions during crime scene processing, although stable during this time period, may not reflect conditions during the entire postmortem period. Conditions may have been altered during discovery of the body, such as by opening doors and windows, removing or adding covers or heating sources. There have also been cases where the presence of many crime scene investigators and installed floodlights in a small room were considered as potential heating sources which may have altered the ambient temperature during crime scene processing [40].

When trying to correct for such alterations retrospectively, both the ambient temperature assumption as well as the cooling speed may have to be altered. Thus, it is of relevance to examine cooling (and potentially also heating) artefacts caused by these post-discovery alterations of a crime scene and their potential influence on temperature-based TSD examination.

Furthermore, a reliable assessment of relevant cooling parameters needs to address the problem of initial room temperatures. Here, exact information or even sufficient rules of thumb to assess the time between the end of the alteration (in the given scenario closing the window) and reversion or approximation to pre-alteration temperature values in case of altered ambient temperature is lacking. Even without alteration, strictly stable and homogenous temperature at typical indoor crime scenes can be considered very rare. Following the thermodynamic principle, air and water form different temperature strata, which may also change over time, especially after manipulations of room openings [41] subject to the potential difference in air temperature (which is in general measured above the ground) between the room and the outside or adjacent rooms [42, 43].

This layering of air may complicate forensic case work, where ambient temperature measurements are usually taken at the height of rectal temperature measurement (i.e., at 0.1 m [1]) and at a certain distance above the ground or corpse [3, 44] (in forensic casework commonly at a height of 1.0 m) to resemble room temperature in general.

This pilot study aims to answer the following questions about a recurring problem in forensic case work:

1. How does opening windows of different sizes for different time intervals during crime scene investigations influence the ambient temperature?
2. Which observation period of ambient temperature profiles is required to ensure reversion or approximation to initial room temperature values after different alteration periods?
3. What are the effects of the respective alterations of the ambient temperature at the crime scene (in this case by opening a window) on the results of Henssge’s method for the estimation of TSD?
4. Is there a benefit for TSD estimation in measuring the ambient temperature at a height of 1.0 m compared to 0.1 m?

## Material and methods

### Ambient temperature

**Fig. 1:**
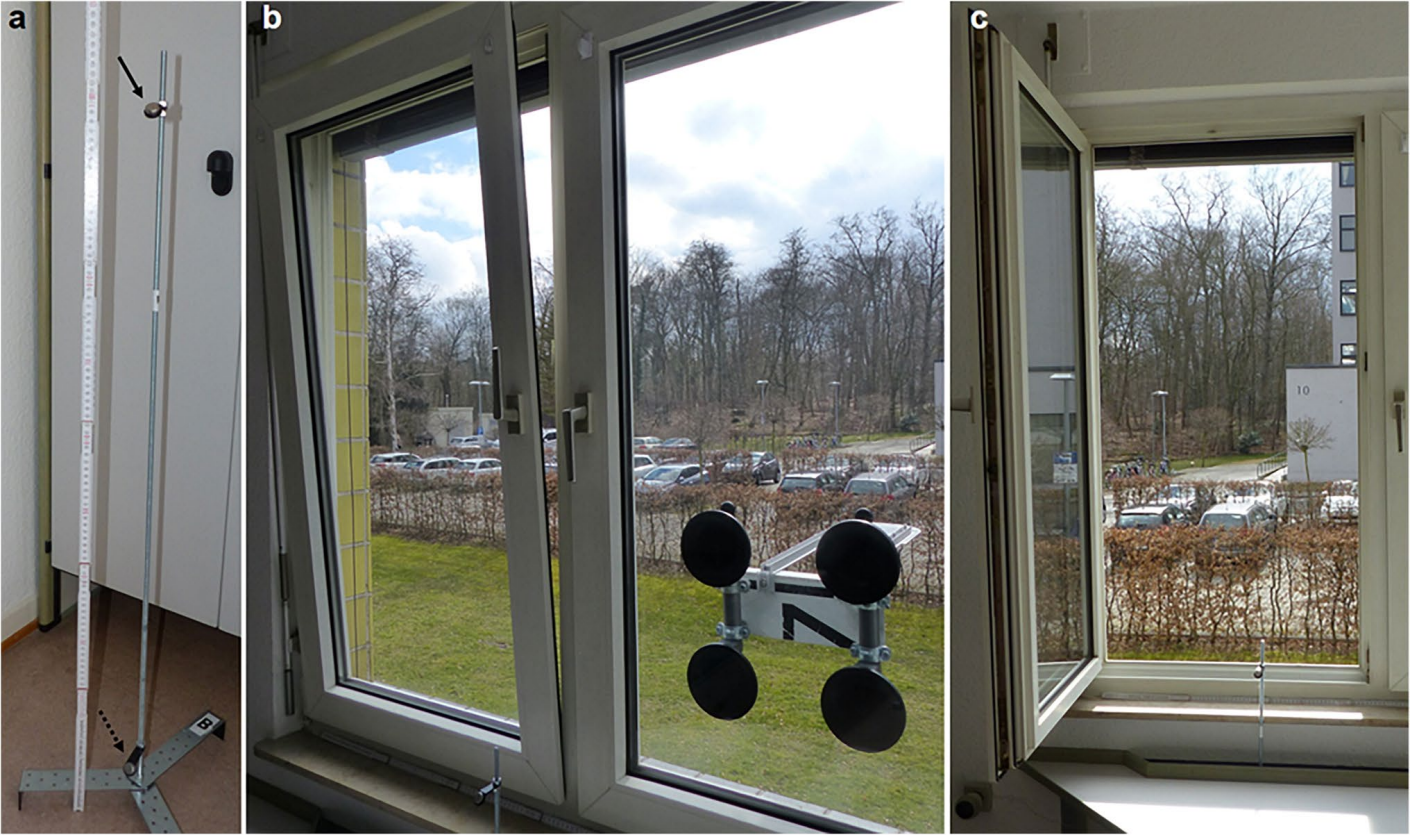
(**a**): Metal rack construction and data logger positioning at 0.1 m and 1.0 m height (black arrow 1.0 m, dotted black arrow 0.1 m). (**b**): Tilt-function exemplary for window a (experimental scenario a1). (**c**): Turn-function exemplary for window a (experimental scenario a2)

**Fig. 2:**
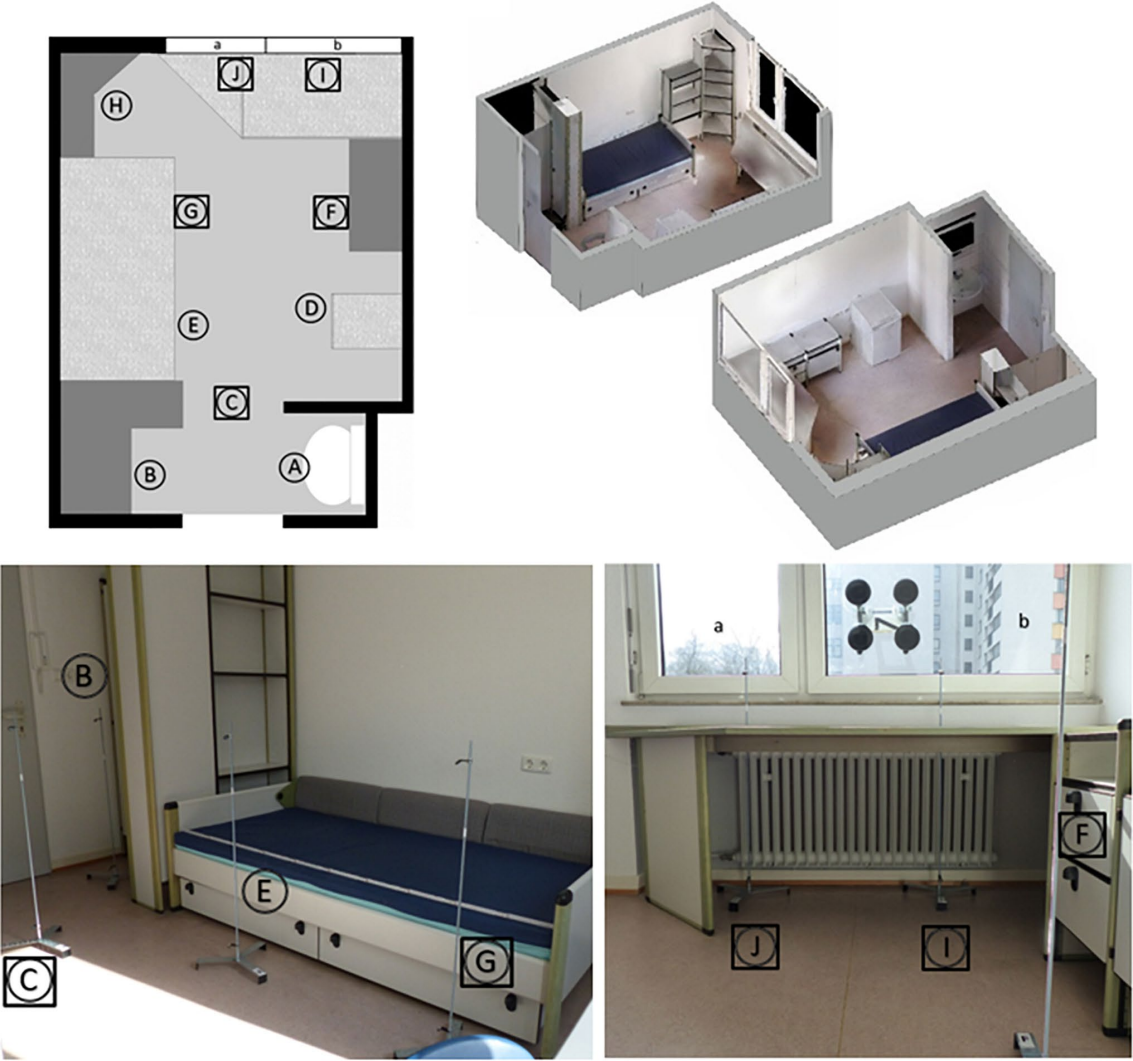
Top left: Ground plan indicating the locations of all indoor data logger pairs (**A**—**J**, squares mark logger pairs included in further statistical analysis). Top right: 3D side views of the apartment. Bottom left: Photograph showing logger pairs **B**, **C**, **E**, and **G**. Bottom right: Photograph showing logger pairs **F**, **I**, and **J** (loggers at a height of 0.1 m positioned under and loggers at a height of 1.0 m positioned above the desk), the radiator location and the south/southeast oriented wall containing windows a and b. Notice the weather station rack attached by vacuum-handles on window b (exemplary setup for testing scenarios a1 and a2)

A furnished studio apartment of approximately 10 m^2^ located on the 2nd floor of a 4-storey apartment building in an urban residential area (Frankfurt/M., Germany) was used, comparable to crime scenes involving social housing, student dormitories, prostitute workplaces, small hotel rooms and the like. This apartment has a split window area consisting of a small tilt and turn window (in the following referred to as “window a”) and a large casement window (in the following referred to as “window b”). Four different experimental scenarios (termed a1, a2, b1, and b2) were tested. Tilt and turn windows, commonly found in some European countries, can be opened in two different ways: For scenario a1 the small tilt and turn window was only partially opened with the upper part of the window tilting into the room and the window base still attached to hinges (Fig. 1, b). For scenario a2, opening of the window much like a door, inwards into the room (Fig. 1, c). Based on this concept, the large, horizontally opening casement window was only partially opened in a simulated tilt function for scenario b1 and completely opened for scenario b2. Accordingly, the following opening surfaces were calculated: 0.18 m^2^ for scenario a1, 0.81 m^2^ for scenario a2, 1.35 m^2^ for scenario b1, and 1.51 m^2^ for scenario b2. In total, the number of experimental window openings amounted to 81 across all four scenarios with window opening periods of 5 (n = 4), 10 (n = 4), 15 (n = 8), 30 (n = 8), 60 (n = 20), 120 (n = 17), 240 (n = 19), and 360 (n = 1) minutes. During the window openings, the ambient temperature was measured using a total of 20 data loggers (iButton® DS1922L, Maxim Integrated Inc., San Jose, CA, USA [45]), attached to a metal rack in pairs of two data loggers per location, one at a height of 1.0 m and one at a height of 0.1 m (Fig. 1, a). The data logger pairs were placed in 10 different locations (A – J) within the apartment furnished with a bed, a desk mounted on the wall, 5 closets and storage racks, one refrigerator (which remained disabled throughout all measurements), and a lavatory (Fig. 2). During the whole measurement period, there were no people in the apartment and the lighting and electronic devices were switched off. The radiator was situated beneath the sill under the desk (Fig. 2, bottom right). The regulator (height above the ground floor approximately 0.7 m) was adjusted to a normal room temperature of approximately 21 °C to resemble the corridor temperature avoiding additional artefacts due to the door opening procedure. The sampling rate of all data loggers was set to one measurement per minute with a resolution of 11-bits (0.0625 °C). The measuring accuracy is specified by the manufacturer as ± 0.5 °C. All data loggers were set with a time delay for the start of temperature data collection after initial synchronization to ensure time-synchronicity in every logger-position. The temperature loggers where delivered in a calibrated state. A loss of calibration due to battery loss did not occur.

### Outside temperature

Since studies comparing temperature measurements at the site of discovery with official data from nearby weather stations showed that the temperature from meteorological weather stations and the actual data can consistently differ significantly regardless of the TSD and the body discovery site [46–49], a dedicated weather station (Gill Multi-plate Radiation Shield [50, 51]) was chosen to yield precise location-based temperature information. It was self-constructed and mounted on a rack attached to the outside surface of the window not used in the respective experimental scenario (Fig. 2, bottom right). Measurements of the outside ambient temperature were executed at the level of the window (> 2 m above the outside ground level) with a distance between weather station and window plane of approximately 60 cm. To avoid malfunction problems, 2 data loggers (resulting in one pair) were placed inside the station. Furthermore, the data of temperature profiles for the complete experimentation phase of the three closest public weather stations (1420, 1424, and 7341 [52]) were acquired from the DWD (Deutscher Wetterdienst, Offenbach, Germany) and a mean temperature of the hourly temperature values was calculated and compared to our measured data. To achieve a high temperature difference and thus evoke maximum effects, experiments were carried out in the winter months of January and February.

### Analysis

Pretests documenting indoor and outdoor temperatures during the course of the day were carried out for 6 days, after three of which shutters were used to provide for a temperature course without relevant solar irradiation artefacts. The door could be opened towards the apartment and was reached through a heated indoor corridor (with an average temperature of approximately 21 °C).

The measurement data collected by the loggers were transferred to, as well as cropped and plotted with the software program Excel (Microsoft Office Professional Plus 2016, Microsoft Corp., Redmond, WA, USA) after completion of the experiments. Statistical analysis was carried out using the statistical software packages SPSS (SPSS Statistics 29, IBM Corp., Armonk, NY, USA) and MedCalc (MedCalc Software Ltd., Ostende, Belgium).

For in total 22 data loggers (10 indoor pairs, location A – J, and 1 outdoor pair) per 81 scenarios (a1, a2, b1, b2) resulting in n = 1,782 recorded temperature data sets (one data set consisting of a varying amount of data points depending on the window opening period). All recorded measurements were reviewed to identify and characterize outliers and common characteristics. Based on these observations and analyses, three optimal locations of data logger pairs for evaluation of temperature profiles of the room for each window were identified. Further analyses were based on data originating from data logger pairs of these three selected locations C, G, and J for scenario a1 and a2 and C, F, and I for scenario b1 and b2 (Fig. 2, top left, bottom left, bottom right).

To determine the expected minimum and maximum ambient temperature alterations and the resulting errors possibly modifying temperature-based TSD estimations, the mean temperature loss within the first 15 min after opening the window and until the lowest recorded temperature for all scenarios were calculated. Furthermore, the respective temperature losses were compared to the window opening surfaces.

The observation period of ambient temperature after closing the windows was assessed. The time span necessary for reversion to pre-alteration temperatures was analyzed for individual temperature data sets with follow-up periods long enough to reverse. Furthermore, we aimed at detecting possible overcompensation phases after reaching reversion levels due to the radiator thermostat response to alteration-induced cooling of the room.

To evaluate the possible influence of the measured temperature alterations on the temperature-based TSD estimation we calculated differences of mean TSD estimations for all tested scenarios. At first, assuming a relatively stable room temperature of 21 °C, the TSD of an unclothed fictional corpse (Henssge empiric correction factor of 1.0) with a weight of 70 kg (serving as the cooling weight) was calculated [53] with presumed rectal body temperature measurements between 36.2 °C and 22.2 °C in intervals of 1.0 °C (resembling a decrease of body temperature over time for typical detection times nearly reaching the initial ambient temperature of 21 °C, bearing in mind, that the permissible critical Q-value of 0.2, was calculated with 24.24 °C and thus the lowest values would not be used for TSD estimation, whereby such problematic cases are already addressed by an previously published proposed solution [10]) using the Henssge Nomogram method [49]. The calculations were then repeated, using the mean values of the measured minimum room temperatures for scenarios a1, a2, b1, and b2 instead of 21 °C and the results were compared.

## Results

### Influence of window opening on ambient temperature

Initial analysis of temperature logger data sets measuring immediately in front of the windows a and b as well as the radiator showed artificially increased temperature losses with an average of 0.39 °C/min (SD 0.53) for the two loggers positioned at “J” and 0.73 °C/min (SD 0.57) at “I”. Despite the immediate drop in temperature after opening the window, the minimum ambient temperature was not reached within the first 10 min after window opening, except in 7 out of 146 recorded temperature data sets with increased temperature loss (logger locations I, J). Therefore, data yielded from these locations (I, J) and with window opening intervals of less than 15 min were excluded, leaving n = 292 temperature data sets consisting of n(a1) = 56 (logger pairs C and G, 14 times scenario a1), n(a2) = 84 (logger pairs C and G, 21 times scenario a2), n(b1) = 72 (logger pairs C and F, 18 times scenario b1), n(b2) = 80 (logger pairs C and F, 20 times scenario b2) for further analysis.

**Fig. 3:**
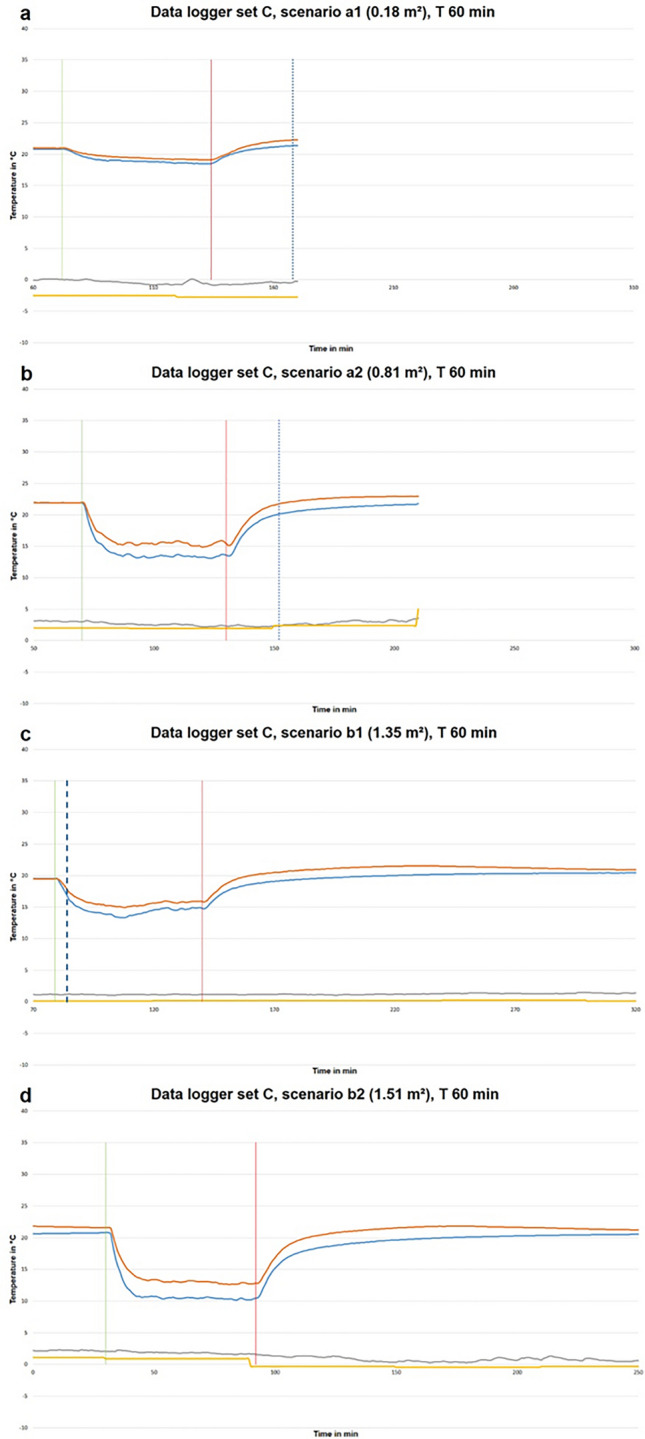
Exemplary temperature curves for data logger pair C of window a tilted (**a**) and entirely opened (**b**) and window b tilted (**c**) and entirely opened (**d**) on opening periods of 60 min. Note, that the shown measurements are not based on the same test day (blue: temperature curve at 0.1 m height; orange: temperature curve at 1.0 m height; grey: mean outdoor temperature profile of the two outside data loggers; yellow: mean outdoor temperature profile of combined weather service data; thin vertical green: window opening; thin vertical red: window closing; dotted blue: sunrise; dashed blue: sunset)

The outside temperature measured during the window opening intervals showed mean values of 2.94 °C (SD 3.26) and a comparison of the curves showed no major deviations from the weather records of the public weather stations (Fig. 3).

**Fig. 4:**
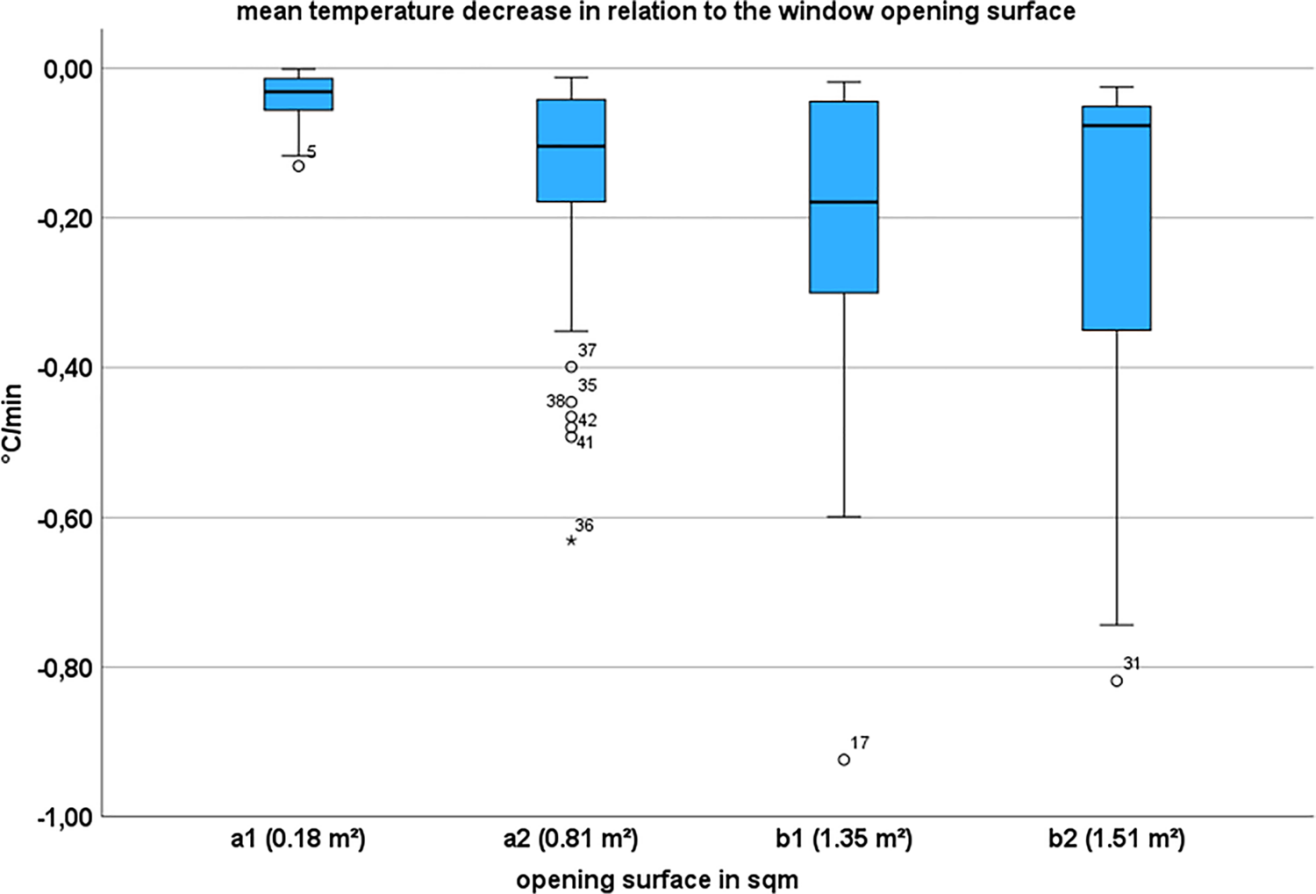
Box plots of the mean temperature decrease rates for the different window opening surfaces including all window opening time intervals

The ambient temperature profiles showed similar patterns for all 292 data sets (Fig. 3). The mean temperature decrease within the first 15 min showed a steep drop of 0.357 °C/min (SD 0.203) while the slope then flattened over the course of time and fell to a mean temperature decrease of 0.004 °C/min (SD 0.021) after 15 min until the window was closed. Statistical analysis revealed a significant difference of these mean temperature decreases using a paired t-test (p-value of < 0.01 and a Cohen’s d of 1.826). The temperature loss varied depending on the window opening surface (Fig. 4). Scenario a1 showed the flattest curves overall with the smallest temperature decrease both within the first 15 min and afterwards in comparison to the other scenarios (Table 1). The absolute temperature differences between the initial ambient temperature and the measured minimum temperature during window opening showed the following mean values: 1.17 °C (SD 0.76) for scenario a1, 6.61 °C (SD 2.59) for scenario a2, 7.52 °C (SD 2.11) for scenario b1 and 9.18 °C (SD 1.81) for scenario b2.

**Table 1:** Values of the minimal, maximal, and mean temperature change rates [°C/min] for the tested scenarios comparing the temperature decrease rates within the first 15 min of window opening with the residual opening time (varying between 15 and 345 min)

		n	max	min	mean	SD
until T15	a1	56	-0.13	0.00	-0.05	0.03
	a2	84	-0.72	-0.10	-0.34	0.13
	b1	72	-0.85	-0.17	-0.41	0.14
	b2	80	-0.84	-0.23	-0.53	0.13
after T15	a1	48	-0.04	0.05	0.00	0.02
	a2	76	-0.07	0.14	0.00	0.03
	b1	64	-0.04	0.02	-0.01	0.02
	b2	72	-0.06	0.06	0.00	0.02

Temperature change in °C/min

### Initial room temperature

For 200 of the further analyzed temperature data sets, the recorded subsequent observation time interval was at least equal to the window opening interval. For the other recordings, these time intervals could not be maintained due to technical reasons. A deviation of ± 0.5 °C was considered as a feasible approximation of initial room temperature.

Scenario a1 was the only one where the temperature differences during window opening were occasionally so small that after the window was closed, the approximation was reached in less than a minute in 12 of the 14 repetitions. For all scenarios, the initial room temperature was approximated within 45 min if the window opening interval was 30 min or less and the subsequent observation period was sufficiently long. Overall, 29 temperature data sets showed an increase above the initial ambient temperature. In these cases, the window opening intervals were always 60 min or longer and the temperature values measured during the subsequent observation interval consistently remained above the initial room temperature by more than 0.5 °C after a short transition period. In 9 temperature data sets, approximation intervals of twice to three times the window opening intervals were observed. A total of 69 temperature data sets did not reach an approximation within the recorded time intervals.

### Influence on the outcome of the TSD estimation

**Fig. 5:**
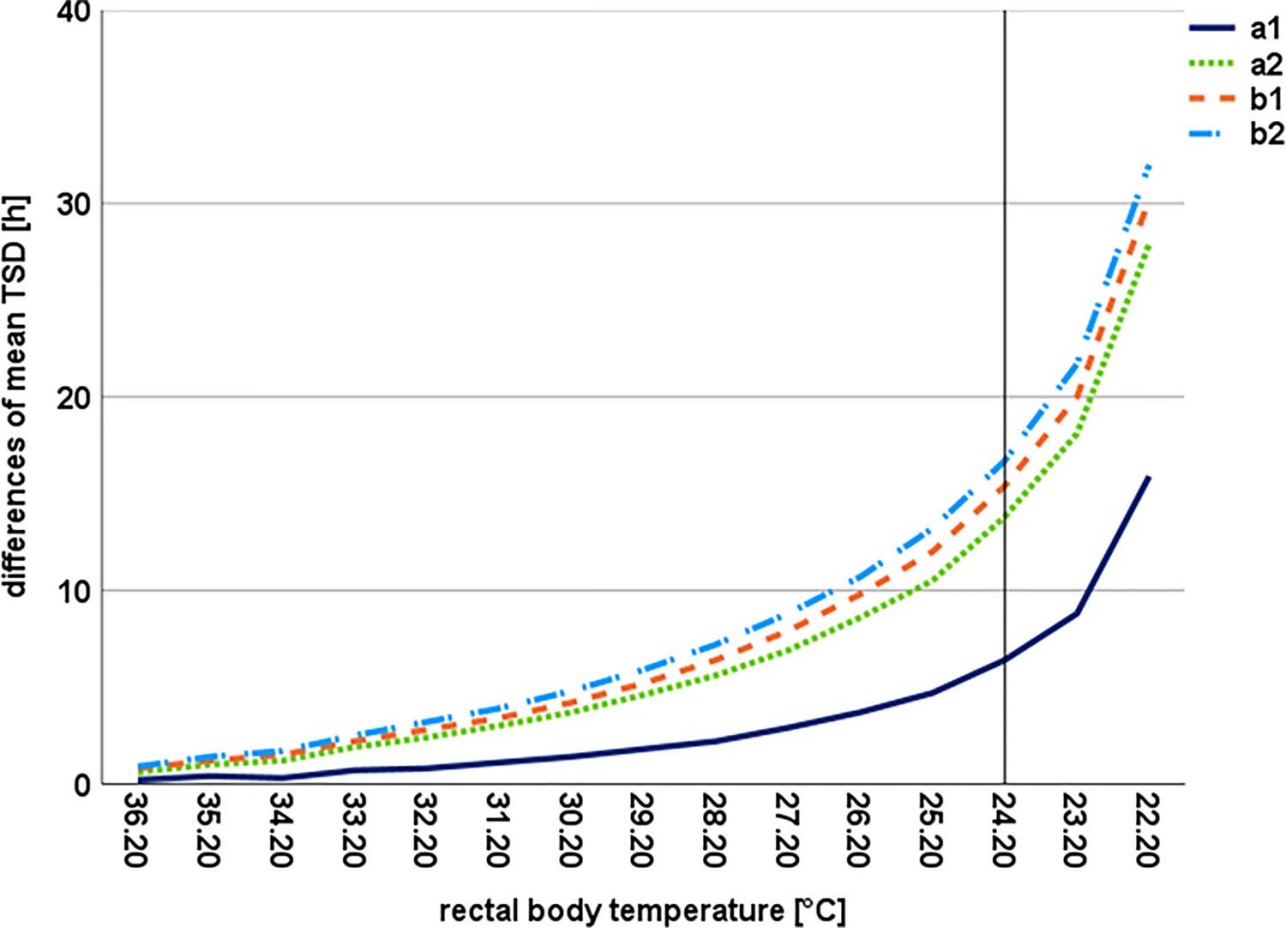
Differences of the calculated fictional mean TSDs due to an alteration of the room temperature (violett continous: curve progression scenario a1; light green dotted: curve progression scenario a2; orange dashed: curve progression scenario b1, light blue dotted and dashed: curve progression scenario b2). Note, that the critical Q-value of 0.2 in the fictional body cooling at a room temperature of 21.0 °C is calculated at 24.24 °C (displayed by the grey vertical line), where a further death time estimation is inadmissible utilizing the nomogram method

Across all further analyzed temperature data sets, respectively for the four scenarios, the values of the room temperature notionally altered by the measured mean minimum temperature data was 19.43 °C for a1, 14.45 °C for a2, 12.87 °C for b1, and 10.44 °C for b2. The mean TSDs were calculated (Table 2) and the differences in hours between the mean TSDs at 21 °C and the mean TSDs altered by the mean minimum temperature data were determined for each scenario and presumed rectal temperature, respectively (Fig. 5). The results showed that the deviations increased as the corpse cools down. While the differences in the calculated mean TSD at a rectal temperature of 35.2 °C ranged from 0.4 h for scenario a1 to 1.4 h for scenario b2, differences between 3.7 h and 10.7 h were noted for a rectal temperature of 26.2 °C. Since only the ambient temperature was measured, a possible change in the cooling pattern of the corpse was *not* taken into account.

**Table 2:** Continuous decrease of fictional rectal temperatures (column 1) and the calculated mean time since death values [hours] according to the Henssge nomogram-method [14] during the course of the fictional body cooling (column 2 assumably unaltered, column 3–6 fictional alteration of the room temperature through the measured mean minimums for scenarios a1, a2, b1, and b2)

rectal temp	Mean TSD [h]	Mean TSD [h]	Mean TSD [h]	Mean TSD [h]	Mean TSD [h]
in °C	initial (21.00 °C)	a1 (19.43 °C)	a2 (14.45 °C)	b1 (12.87 °C)	b2 (10.44 °C)
36.2	3.40	3.20	2.80	2.60	2.50
35.2	5.00	4.60	4.00	3.80	3.60
34.2	6.30	6.00	5.10	4.80	4.60
33.2	8.00	7.30	6.10	5.80	5.50
32.2	9.60	8.80	7.20	6.80	6.40
31.2	11.20	10.10	8.20	7.80	7.30
30.2	13.00	11.60	9.30	8.80	8.20
29.2	15.00	13.20	10.40	9.80	9.10
28.2	17.20	15.00	11.60	10.80	10.00
27.2	19.80	16.90	12.90	11.90	11.00
26.2	22.80	19.10	14.20	13.00	12.10
25.2	26.30	21.60	15.80	14.30	13.10
24.2	31.00	24.60	17.20	15.60	14.30
23.2	37.00	28.20	18.90	17.00	15.30
22.2	48.80	32.90	20.90	18.60	16.80

### Measuring positions

A comparison of the ambient temperature recorded by the data loggers at a height of 0.1 m and 1.0 m revealed significant differences. The measurements recorded at a height of 0.1 m showed a mean temperature loss of 0.38 °C/min (SD 0.21) within the first 15 min after window opening as compared to 0.33 °C/min (SD 0.20) at a height of 1.0 m. For the further temperature profile after 15 min until closing of the window the mean temperature loss was around 0.0 °C/min regardless of the measurement height (0.001 °C/min with SD 0.024 at 0.1 m and 0.007 °C/min with SD 0.018 at 1.0 m height). The graphs (Fig. 3) reflect the calculated values with a steeper temperature decrease within the first 15 min for measurements at a height of 0.1 m in contrast to the measurements at 1.0 m. During the complete course of the decrease, plateau phase, and increase to reversion values, there was no intersection between 0.1 m-graphs and 1.0 m-graphs in any of the experiments. For the room conditions in our study this resulted in slightly lower temperature differences between the initial ambient temperature and the temperatures measured at a height of 1.0 m. Even when measuring the ambient temperature before a window was opened, differences in the mean measured room temperatures at different heights were seen due to thermodynamic effects. In our experiments, the initial non-altered mean ambient temperature values at a height of 0.1 m were 20.65 °C (SD 1.06) and 21.42 °C (SD 1.01) at a height of 1.0 m while the regulator setting was approximately 21 °C.

## Discussion

In the present pilot study, we examined ambient temperature effects of scene alterations due to the opening of windows of different sizes including different opening modes (scenarios a1, a2, b1, and b2) for various periods of time on a simulated crime scene consisting of a furnished 10 m^2^ apartment. We analyzed temperature data from selected points in the room at two different heights as well as the outside temperature recorded during our experiments. In addition to the influence on the ambient temperature and the time required to re-approximate the initial room temperature after the room had been cooled down, we examined a possible influence of a change in ambient temperature on the calculated TSD and reviewed the impact of standardized temperature measurements at a heights of 0.1 m and 1.0 m for crime scenes.

While there are thermodynamic relevant constructional properties of different building and/or rooms, the chosen apartment can be assumed to be representative for indoor crime scenes, involving e.g. social housing, student dormitories, prostitute workplaces, small hotel rooms and the like, which is not rare in forensic case work in Europe. Furthermore, internal and external sources of heat or cooling—such as electrical appliances, the actual heating system installed, lighting equipment, or the presence of people radiating body heat during any temperature measurement—must be taken into account as potential influencing factors [40, 44]. Therefore, the results originating from this study may serve to illustrate typical cooling behavior and the respective temperature profiles after scene manipulation by opening a window in this specific case, but were *not* intended and may not serve as a mathematical model for the calculation of cooling curves in other specific room settings. While regression formulas were calculated for a variety of temperature profiles, we chose to not include these in the present manuscript to avoid case-related calculation of temperatures and time spans based on values that are room-specific for the present setting. In this respect, a combination of simulation methods (e.g., thermodynamic and finite element modelling) using 3D scans of crime scenes, could provide a model applicable for case-specific individualization in the future, as sudden changes in ambient temperature may also be integrated into the simulations [23, 54]. It is also important to mention that our pilot study took place in winter with mean values of the outside temperature of 2.94 °C (SD 3.26) during the recorded experiments.

The evaluation of the temperature profiles showed that in small rooms, the complete opening of a small window (scenario a2) or the incomplete opening of a large window (scenario b1), and even more the complete opening of a large window (scenario b2) for a period of only 15 min may have a significant effect on the ambient temperature value to be used in TSD calculations, which is relevant for determining the TSD. The incomplete opening of the small window (scenario a1) had the least influence on the room temperature. Therefore, once this time period has been reached, most of the temperature loss has already occurred. Reversion or approximation to initial room temperature values after different alteration periods could only be determined for 194 out of 292 temperature data sets as in some cases the subsequent observation interval was not long enough or there was an overcompensation due to thermostat induced excess beyond the initial room temperature was exceeded. With opening times of 60 min or more for the scenarios a2, b1 and b2, it occasionally took twice to three times the opening time to ensure an approximation of the initial temperature of up to 0.5 °C after closing the window. Longer subsequent observation periods would have been necessary in order to reconstruct the reversion or approximation to initial room temperature values even in the event of overcompensation.

For TSD calculations using the Henssge Nomogram method with set parameters as mentioned above, the assumed initial ambient temperature of 21 °C for the first calculations was later modified by the mean values of the measured minimum room temperature for each scenario. It was noticeable that the deviations depending on the assumed ambient temperature in the calculated TSD increased as the rectal temperature decreased, which has been described before [44]. In addition, if there is a drop in temperature, assuming the correct ambient temperature is crucial. For example, if a corpse has been in an ambient temperature of 21 °C for a longer period of time and the rectal temperature has already dropped to 21 °C when measured by the forensic pathologist or a criminal investigator after his arrival but a window was opened during the discovery of the body and the room has cooled down by the time the measurements are taken, this could lead to erroneous TSD calculations based on the false assumption that the corpse has not yet cooled down to the ambient temperature and has not yet reached critical Q (0.2). This illustrates the importance to consider the limitations of the nomogram method in forensic casework, as the selection of the potentially erroneously measured ambient temperature determines the applicability of the method itself, as case reports have already shown [40].

The comparison of the ambient temperature readings recorded by the data loggers at different measuring heights revealed that there were already differences in the values for the initial room temperature when measured at a height of 0.1 m or 1.0 m. The measurements at a height of 1.0 m were subject to relatively smaller deviations before and after the window opening (due to the thermodynamically induced vertical temperature distribution and layer formation in the room) as compared to the measurements at a height of 0.1 m and re-approximated to their initial values more rapidly, so that the measurement position at a height of 1.0 m seems more robust and less susceptible to artifacts. Whereas, at first, it seems to be reasonable to measure the ambient temperature at a height of 1.0 m at the same time as measuring the rectal temperature of the corpse, this procedure does not appear to be very useful at a second glance. As the measurements taken before window opening demonstrate, the temperature directly affecting the corpse can differ from the temperature at a height of 1.0 m even without changes in the environment. In addition, despite a relatively more stable temperature profile, measurements at a height of 1.0 m also showed a noticeable temperature drop. It therefore appears to be preferable to monitor the room temperature for a prolonged time period and to perform the TSD calculations with the adjusted values. One possibility that needs to be tested in further studies would be to place data loggers at the same height as the corpse, e.g. for 24 h after restoring the initial conditions of the room (for our study: closing the window). If the body should be still at the crime scene, the temperature measurements could be performed with a horizontal distance of 1.0 m and at the vertical height of the body (e.g. 0.1 m) to avoid possible radiation effects from the body itself [55]. In this respect, it is crucial to consider, that constructional properties (heating pipes passing underneath the floor, e.g. floor heating) may cause erroneous ambient temperature values, if measured at a height of 0.1 m. To prevent such additional artifacts measuring the ambient temperature at an indoor crime scene, the application of a thermal imaging camera could be beneficial.

## Recommendations for practical case work

Based on our results, we would like to raise awareness for crime scene alteration-induced effects on the ambient temperature value for calculating the TSD. After identifying that a change in the initial conditions has taken place, the aim should be to determine the original temperature affecting the corpse and its cooling process. Our recommendation, in line with previous recommendations of other authors [44], is to restore the initial conditions as quickly as possible and then place data loggers at the height and location of the corpse, if possible supported by additional data loggers at a sufficient distance from windows and heat sources for at least 24 h to identify regular room temperature influences over the course of the diurnal rhythm. If the exact opening time remains controversial, an interdisciplinary reconstruction effort might be helpful, preferably taking into account comparable outdoor conditions (e.g. similar outdoor temperature and other weather conditions). In order to improve the precision of any reconstruction efforts, it is also advisable to document any crime scene alterations and possible artefacts relevant for a time since death estimation in detail [44]. Moreover, we endorse the use of temperature loggers in research and forensic casework in general, as they are affordable, small, easy to use and exhibit a reasonable resolution and measurement accuracy, so that their placement does not significantly disrupt indoor crime scene processing. In forensic entomology, temperature loggers have been established for some time and provide valid data [46, 56, 57].

## Limitations

In addition to the limitations already mentioned in the discussion section regarding the building and observation periods, there were some further limitations to this pilot study. When assessing the TSD calculations, one major limitation in our pilot study was that only the ambient temperature was measured, a possible change in the cooling pattern of a corpse was not taken into account. However, with evident temperature differences and longer exposure times, at least longer than 1 to 2 h (accordingly to Henssge and Madea [44]), it must be assumed that the cooling of the corpse changes, so that further studies, i.e., with cooling models, are required to examine changes in rectal temperature measurements, especially since it as been shown, that a sudden change of ambient temperature for a substantial amount of time may accelerate or slow down the temperature drop [26, 27] (particularly in skin temperature based models [58]) or even form a hardly assessable second temperature plateau [33].

## Data Availability

The original data are available (upon request) from the corresponding author.

## Declarations

### Human and animals participants

The research presented in this study did not include human participants or animals.
